# COVID-19 in an Adult with Down Syndrome: Impact on Autoimmune Response

**DOI:** 10.1155/2022/6128496

**Published:** 2022-04-13

**Authors:** Hiroshi Kobayashi, Mana Akiniwa, Yoshiko Yamaguchi, Yuji Hirai, Akiko Aoki

**Affiliations:** ^1^Department of Rheumatology, Tokyo Medical University Hachioji Medical Center, 1163 Tatemachi, Hachioji, Tokyo 1930998, Japan; ^2^Department of General Medicine and Primary Care, Tokyo Medical University, Hachioji Medical Center, 1163 Tatemachi, Hachioji, Tokyo 1930998, Japan; ^3^Department of Infectious Diseases, Tokyo Medical University, Hachioji Medical Center, 1163 Tatemachi, Hachioji, Tokyo 1930998, Japan

## Abstract

We here report a case of COVID-19 with effusion prior to the development of pneumonia in an adult with Down syndrome. Serositis due to rheumatic disease was initially suspected because of a high titer of serum autoantibodies and leukocytopenia; however, SARS-CoV-2 infection was confirmed by reverse transcription polymerase chain reaction on admission after previous negative tests. Several cases of COVID-19 have been associated with autoimmune responses along with some cases of COVID-19 with autoimmune manifestations. Furthermore, patients with Down syndrome have a higher mortality risk from COVID-19 than the general population, and it is believed that a high sensitivity to the interferon response may contribute to the increased severity of the disease. Thus, careful attention should be paid to autoimmune manifestations due to SARS-CoV-2 infection for ensuring a proper and timely diagnosis, especially in patients with Down syndrome.

## 1. Introduction

Coronavirus disease 2019 (COVID-19) is an emerging infectious disease caused by severe acute respiratory syndrome coronavirus 2 (SARS-CoV-2) that causes interstitial pneumonia with or without respiratory failure and thrombosis due to hypercoagulability [1]. The host immune responses to SARS-CoV-2 infection are associated with a variety of autoimmune features, and autoantibody production has also been observed in patients with COVID-19 [2]. Indeed, there are some rare cases with features of systemic lupus erythematosus (SLE) following COVID-19 [3, 4]. However, the clinical significance of the autoimmunity triggered by SARS-CoV-2 infection remains unclear.

The high mortality risk of COVID-19 in individuals with Down syndrome has been reported, which is considered related to the immune disorder associated with the syndrome [5–7]. Down syndrome, a trisomy of chromosome 21, is associated with comorbidities of congenital heart disease, respiratory infections, and chronic immunomodulatory disorders [8]. Interferon (IFN) receptor genes, which are located on chromosome 21, are overexpressed in patients with Down syndrome, suggesting that hypersensitivity to IFN stimulation in response to infection induces excessive cytokine production by immune cells, leading to a cytokine storm [5].

Here, we report an interesting case of COVID-19 with effusion and autoantibody production prior to the development of pneumonia in an adult with Down syndrome.

## 2. Case Presentation

A 44-year-old woman with Down syndrome developed a sudden fever of approximately 38°C in the middle of March 2021, corresponding to the fourth wave of the COVID-19 epidemic in Japan. She had an atrial septal defect surgery eight years previously and was living with her father and doing well in her daily life.

**Figure 1:**
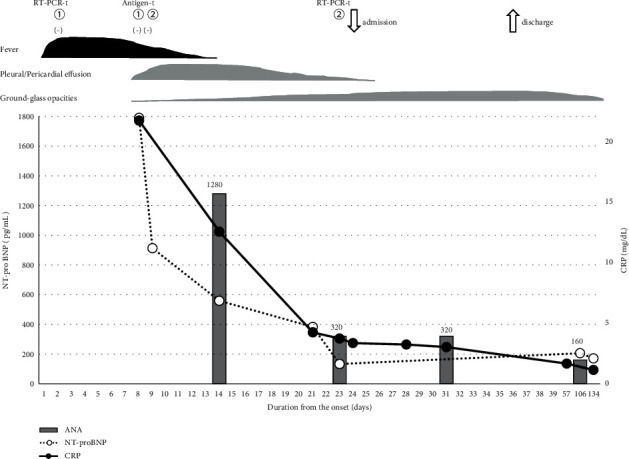
Clinical course from the day of symptom onset. The clinical symptoms and changes in N-terminal pro-B type natriuretic peptide (NT-pro-BNP), C-reactive protein (CRP), and anti-nuclear antibody (ANA) are shown. The number at the top of the antinuclear antibody indicates the titer. RT-PCR-t means reverse transcription-polymerase chain reaction test for severe acute respiratory syndrome coronavirus 2 (SARS-CoV-2). Antigen-t means antigen test for SARS-CoV-2. The numbers indicate the order in which the tests were performed.

**Figure 2:**
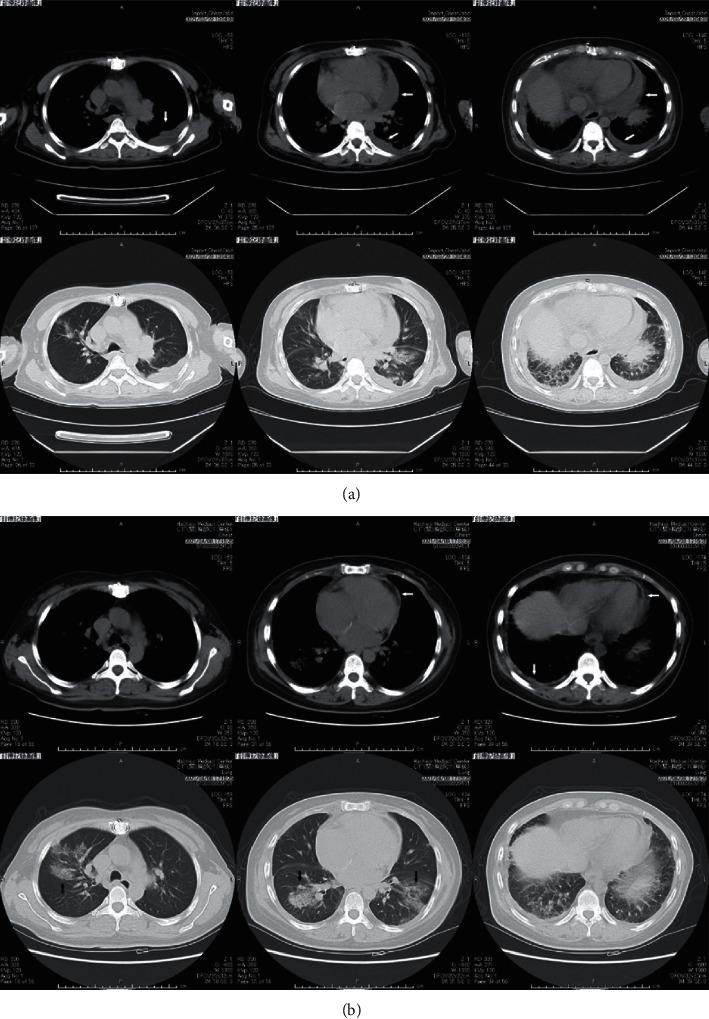
Chest plain computed tomography scan image performed at the referral hospital (a) at the time of admission to our hospital (b). The white arrows indicate the accumulated pleural effusion and pericardial fluid, or thickened pleura and pericardium. The black arrows indicate exacerbated ground-glass opacities.

The clinical course from fever onset is shown in Figure 1. The patient was tested for SARS-CoV-2 by reverse transcription polymerase chain reaction (RT-PCR) from a nasal swab, which was negative, and was prescribed antibiotics at a clinic because she had been febrile for one week. She was examined further at a neighborhood hospital. She was tested for SARS-CoV-2 by a nasopharyngeal antigen test twice, which was negative both times. A chest computed tomography (CT) image showed bilateral pleural effusion, which was more severe on the left side, and pericardial effusion (Figure 2(a)). Since blood tests showed a high serum level of N-terminal pro-B type natriuretic peptide (NT-pro-BNP) of 1790 pg/mL, the reference value (r.v.) of NT-pro-BNP is 125 pg/ml, she was prescribed diuretics. She had no arrhythmia and echocardiography showed no left ventricular dysfunction. However, the titer of serum anti-nuclear antibody was high (1/1280, homogenous and nucleolar pattern, the reference range is <1/40) and rheumatoid factor was positive (42 IU/mL, r.v. <18 IU/mL) with an increased erythrocyte sedimentation ratio (114 mm/h, r.v. 20 mm/h). Since the patient had a familial predisposition to rheumatoid arthritis, rheumatic disease was suspected as the cause of the pleural and pericardial effusions, and she was referred to our outpatient clinic on the 23^th^ day after symptom onset.

A new chest CT image showed exacerbation of patchy ground glass opacities in the bilateral lung fields, but the pericardial effusion had almost disappeared while leaving thickening of the pleura and pericardium (Figure 2(b)). At this point, the nasopharyngeal swab RT-PCR for SARS-CoV-2 was positive, and therefore the patient was diagnosed with COVID-19 and admitted to our hospital.

She had a facial appearance typical of Down syndrome with mild erythema. Although she had slight difficulty in communicating because of her intellectual disability, she was clearly conscious. The vital signs were stable on physical examination: height was 151 cm, weight was 51 kg, body temperature was 36.6°C, blood pressure was 106/65 mmHg, SpO_2_ was 94% (room air), and the respiratory rate was 18 breaths/minute. The laboratory data on admission are shown in Table 1. The serum C-reactive protein level and erythrocyte sedimentation rate were elevated with mild lymphopenia (leukopenia was also confirmed subsequently) and anti-nuclear and anti-cardiolipin antibody tests were positive, whereas the rheumatoid factor was normal. Venous ultrasonography of the lower extremities was performed because of elevated D-dimer levels, but deep vein thrombosis was not indicated. Infectious pathogen screening tests did not detect any pathogens other than SARS-CoV-2.

After 12 days of follow-up, the patient was discharged without the use of glucocorticoids or antiviral drugs. She required low doses of oxygen inhalation due to mild hypoxemia at night. After discharge, she was fine, but developed fibrotic changes in both lower lungs with an elevated serum Krebs von den Lungen-6 level (maximum 1307 U/mL, r.v. <500 U/mL). The serum anti-nuclear titer (1/160) and anti-cardiolipin antibody level (20 U/ml, r.v. <10 U/mL) were decreased as clinical symptoms improved.

## 3. Discussion

We experienced a case of COVID-19 with effusion and autoantibody production prior to pneumonia development in an adult with Down syndrome. As an initial RT-PCR test for SARS-CoV-2 was negative, a differential diagnosis of SLE was made. Indeed, this case fulfilled several of the 2019 European League Against Rheumatism/American College of Rheumatology Classification Criteria for SLE (10 points): fever (2 points), effusion (5 points) and leukopenia (3 points) in addition to a high serum level of anti-nuclear antibody. Upon repeated RT-PCR test that was positive, the definitive diagnosis of COVID-19 was made.

We consider the cause of pleural and pericardial effusion as serositis based on pleural and pericardial thickening with severe inflammation. Of course, the possibility of heart failure could not be completely ruled out without pleural or pericardiocentesis. Although relatively rare, pleuritis and pericarditis have been reported as severe complications in COVID-19. Li et al. reported CT findings associated with severe COVID-19 pneumonia, including 8.4% pleural effusion and 4.8% pericardial effusion [9]. Finn et al. reported that 12.2% of patients with severe cardiac complications of COVID-19 had pericarditis, more than half of whom presented with cardiac tamponade and cardiogenic shock [10].

Similar to the present case, a case of a 21-year-old patient with Down syndrome who developed acute respiratory distress syndrome with SARS-CoV-2 infection and cardiac tamponade due to pericarditis was recently reported [11]. Interestingly, this patient also had complications due to an autoimmune disease causing severe hypothyroidism with anti-thyroid antibodies. Down syndrome has been reported to be associated with a high COVID-19 mortality risk, which is considered associated with the immunomodulatory disorders linked to the syndrome [5–7]. The results from a cohort study of 8 million adults revealed that the adjusted hazard ratio (95% confidence interval) for the association between Down syndrome and death by COVID-19 was 10.39 (7.08–15.23) [6]. Furthermore, in the context of immunomodulatory disorders, the loci for IFN receptors are located on chromosome 21; since this chromosome is in trisomy for Down syndrome, these genes are overexpressed. Therefore, Espinosa proposed a model in which hypersensitivity to IFN stimulation induces excessive cytokine production by immune cells [5]. Moreover, hyperreactivity of the type I IFN pathway, which is linked to SLE pathogenesis, is observed in Down syndrome, and SLE associated with Down syndrome has also been reported [12].

However, the association between COVID-19 and SLE remains unclear. Some SLE cohort reports suggest that the rate of symptomatic COVID-19 in patients with SLE (4%) is higher than that of the general population (2%), but no significant increases in DNA antibodies were associated with infection [13]. By contrast, cases with a lupus-like syndrome or SLE manifestation simultaneously or following COVID-19 have been reported [3, 4]. Infection of certain viruses, including Epstein-Barr virus, cytomegalovirus, parvovirus B19, and some retroviruses, could lead to the development of autoimmune features and autoantibody production, resulting in the so-called “lupus-like syndrome,” or “lupus mimickers” [14, 15]. Notably, a recent study showed that autoantibodies associated with autoimmune diseases such as SLE, scleroderma, or myositis, are identified in approximately 50% of patients following SARS-CoV-2 infection, which may be at least partly associated with the clinical features of COVID-19 [2]. Moreover, Gracia-Ramos et al. describe the characteristics of patients of new-onset systemic lupus erythematosus during or after SARS-CoV-2 infection. Serosal involvement was seen in 4 cases, (pleural effusion 3, pericardial effusion 2, ascites 1), as was observed in our case [16]. As pleuritis is the most common pulmonary complication in patients with SLE but uncommon in COVID-19, its presence is considered to be associated with activity of SLE [17].

In conclusion, this case highlights that careful attention should be paid to autoimmune manifestations due to SARS-CoV-2 infection to ensure making a proper diagnosis of COVID-19, especially in patients with Down syndrome.

## Figures and Tables

**Table 1:** Laboratory data on admission.

	Patient	r.v.
Blood count		
WBC (/mm^3^)	5830	4000–8000
Neutrophils (%)	73.8	40–60
Lymphocytes (%)	17.1	25–45
Eosinophils (%)	1	1–6
Hb (g/dL)	11.5	12–16
PLT (× 10^4^/mm^3^)	34.5	15–35
ESR (mm/h)	120	<20
Blood biochemistry		
TP (g/dL)	8.8	6.6–8.1
Albumin (g/dL)	2.8	4.1–5.1
T-Bil (mg/dL)	0.2	0–0.4
AST (U/L)	28	13–30
ALT (U/L)	22	7–23
AMY (U/L)	79	44–132
ALP (U/L)	87	38–113
LD (U/L)	260	124–222
CK (U/L)	31	41–153
UA (mg/dL)	4.8	2.6–5.5
BUN (mg/dL)	14.7	8–20
Cr (mg/dL)	0.47	0.46–0.79
Na^+^ (mmol/L)	138	138–145
K^+^ (mmol/L)	4.7	3.6–4.8
Cl^−^ (mmol/L)	101	101–108
BS (mg/dL)	93	73–109
KL-6 (U/mL)	406	<500
BNP (pg/mL)	21.2	0–20
Haptoglobin (mg/dL)	313	25–176
phenotype	2–2	
D-Dimer (mg/L)	4.12	<0.50
Serology		
CRP (mg/dL)	3.34	0.00–0.14
Ferritin (ng/mL)	107.9	10–110
CH50 (U/mL)	63.5	30–45
IgG (mg/dL)	3660	861–1747
IgA (mg/dL)	550	93–393
IgM (mg/dL)	107	50–269
RF (U/mL)	15	<18
anti-CCP Ab	0.5	<4.5
ANA (titer)	1: 320	<1: 40
Pattern	Homo/spec/nuc
Anti-CL IgG (U/mL)	31	<10
Anti-DNA Ab	4.5	<6.0
Anti-RNP Ab	<2.0	<10.0
Anti-Sm Ab	2.9	<10.0
Anti-Scl70 Ab	<1.0	<10.0
Anti-SS-A Ab	<1.0	<10.0
MPO-ANCA	1.0	<3.5
Pathogen tests		
SARS-CoV-2 RT-PCR	(+)	(−)
*β*-D glucan	12.2	<20.0
HBs Ag	0.00	<0.05
HCV Ab	0.12	<1.0
HIV Ag/Ab	0.08	<1.00
TB-specific IFN-*γ*	(−)	(−)
Blood culture	(−)	(−)
Legionella Ag	(−)	(−)
St. pneumoniae Ag	(−)	(−)
Urinalysis		
Protein	(−)	(−)
Occult blood	(−)	(−)

Footnote: r.v., reference value; WBC, white blood cells; Hb, hemoglobin; PLT, platelet; ESR, erythrocyte sedimentation ratio; TP, total protein; T-Bil, total bilirubin; AST, aspartate aminotransferase; ALT, alanine aminotransferase; AMY; amylase; LD, lactose dehydrogenase; CK, creatine kinase; UA uric acid; BUN, blood urea nitrogen; Cr, creatinine; Na^+^, sodium level; K^+^, potassium level; Cl^−^ chloride level; BS, blood sugar; KL-6, Krebs von den Lungen-6; BNP, brain natriuretic peptide; CRP, C-reactive protein; CH50, 50% hemolytic unit of complement; Ig, immunoglobulin; RF, rheumatoid factor; anti-CCP; anti-cyclic citrullinated peptide; Ab, antibody; ANA anti-nuclear antibody; homo, homogeneous; spec, speckled; nuc, nucleolar; anti-CL, anti-cardiolipin; anti-DNA, anti-deoxyribonucleic acid; anti-RNP, anti-ribonucleoprotein; anti-Sm, anti-Smith; anti-Scl70, anti-scleroderma70; anti-SSA, anti-Sjögren's-syndrome-related antigen A; MPO-ANCA, myeloperoxidase-anti-neutrophil cytoplasmic antibody; SARS-CoV-2, severe acute respiratory syndrome coronavirus 2; RT-PCR, reverse transcription polymerase chain reaction; HBs; hepatitis B surface; Ag, antigen; HCV, hepatitis C virus; HIV, human immunodeficiency virus; TB, tuberculosis; IFN, interferon; St. pneumoniae, Streptococcus pneumoniae.

## Data Availability

No data sets were used other than the patient's medical record.
